# Current genetic diagnostics in inborn errors of immunity

**DOI:** 10.3389/fped.2024.1279112

**Published:** 2024-04-10

**Authors:** Sandra von Hardenberg, Isabel Klefenz, Doris Steinemann, Nataliya Di Donato, Ulrich Baumann, Bernd Auber, Christian Klemann

**Affiliations:** ^1^ Department of Human Genetics, Hannover Medical School, Hannover, Germany; ^2^ Department of Pediatric Pneumology, Allergology and Neonatology, Hannover Medical School, Hannover, Germany; ^3^ Department of Pediatric Immunology, Rheumatology and Infectiology, Hospital for Children and Adolescents, University of Leipzig, Leipzig, Germany

**Keywords:** inborn errors of immunity, next-generation sequencing, technologies in genetic diagnostics, variants of unknown significance, genes of unknown significance

## Abstract

New technologies in genetic diagnostics have revolutionized the understanding and management of rare diseases. This review highlights the significant advances and latest developments in genetic diagnostics in inborn errors of immunity (IEI), which encompass a diverse group of disorders characterized by defects in the immune system, leading to increased susceptibility to infections, autoimmunity, autoinflammatory diseases, allergies, and malignancies. Various diagnostic approaches, including targeted gene sequencing panels, whole exome sequencing, whole genome sequencing, RNA sequencing, or proteomics, have enabled the identification of causative genetic variants of rare diseases. These technologies not only facilitated the accurate diagnosis of IEI but also provided valuable insights into the underlying molecular mechanisms. Emerging technologies, currently mainly used in research, such as optical genome mapping, single cell sequencing or the application of artificial intelligence will allow even more insights in the aetiology of hereditary immune defects in the near future. The integration of genetic diagnostics into clinical practice significantly impacts patient care. Genetic testing enables early diagnosis, facilitating timely interventions and personalized treatment strategies. Additionally, establishing a genetic diagnosis is necessary for genetic counselling and prognostic assessments. Identifying specific genetic variants associated with inborn errors of immunity also paved the way for the development of targeted therapies and novel therapeutic approaches. This review emphasizes the challenges related with genetic diagnosis of rare diseases and provides future directions, specifically focusing on IEI. Despite the tremendous progress achieved over the last years, several obstacles remain or have become even more important due to the increasing amount of genetic data produced for each patient. This includes, first and foremost, the interpretation of variants of unknown significance (VUS) in known IEI genes and of variants in genes of unknown significance (GUS). Although genetic diagnostics have significantly contributed to the understanding and management of IEI and other rare diseases, further research, exchange between experts from different clinical disciplines, data integration and the establishment of comprehensive guidelines are crucial to tackle the remaining challenges and maximize the potential of genetic diagnostics in the field of rare diseases, such as IEI.

## 1 Introduction

Today's standard of care for patients with clinically diagnosed or suspected inborn errors of immunity (IEI) involves genetic testing. The clinical characterization and the selection of the testing method influences the probability of obtaining a molecular diagnosis. In general, several genetic testing methods are available, including Sanger sequencing of single genes, targeted gene sequencing panels (targeted Next Generation Sequencing, tNGS), whole exome sequencing (WES), and whole genome sequencing (WGS), which can all be expanded to trio- or whole-family analyses. The choice of method largely depends on the clinical presentation, the suspected type of IEI, and the access to resources (1). When the patient's symptoms closely match a specific type of IEI, targeted gene panels that test a set of selected genes known to be associated with IEI, can be a quick and cost-effective first-line method (1). On the other hand, when the clinical presentation is less specific, or when initial targeted gene testing was inconclusive, more comprehensive methods such as WES or WGS may be employed. The goal of genetic testing in IEI is not only to confirm the clinical diagnosis but also to improve patient management. A genetic diagnosis can inform about prognosis, guide treatment decisions, enable genetic counselling, and provide the opportunity for predictive family testing for relatives at-risk. Patient characteristics, including their phenotype, family history, and ethnicity, can also influence the selection of the testing method and thus the likelihood of obtaining a diagnosis. This review aims at providing a summary for the clinician of the current genetic diagnostic tools available in the clinics (tNGS, WES, WGS). In addition, we provide an outlook on the more elaborative tools such as RNAseq, epigenetics and proteomics and used widely on research basis today to facilitate the diagnosis of IEI.

### 1.1 Milestones in developments

The history of genetic diagnostics began in the 20th century with the advent of technologies that allowed scientists to isolate and understand the structure of DNA. The first breakthrough was the discovery of the double helix structure of the DNA in 1953 that paved the way for the field of molecular genetics (2). Cytogenetic techniques visualized chromosomes and found abnormalities, starting clinical genetic diagnostics. Fluorescence *in situ* hybridization (FISH) improved detection of chromosome rearrangements. The chain-termination method developed by Frederick Sanger in 1977 revolutionized DNA sequencing for genetic diagnostics (3). It is primarily used to analyze known disease-associated genes or genomic regions linked to patient symptoms. Sanger sequencing was the method of choice for decades used mainly for identifying single nucleotide variants (SNVs) and is still considered for the validation of variants identified by other sequencing methods. However, the method´s main limitation is that it can only sequence a comparatively small number of bases at a time, making it less suitable for conditions that can be caused by variants in different genes, as is the case for many IEIs. It detects SNVs or small insertions/deletions but struggles with larger structural genomic variations, like extensive deletions or duplications of whole exons. Multiplex ligation-dependent probe amplification (MLPA) is used alongside Sanger sequencing to address these genomic variants. Apart from SNVs, Array-based comparative genomic hybridization (array-CGH) (4), introduced in the 1990s, expedited identifying deletions, duplications, and unbalanced translocations.

The collaborative international Human Genome Project, launched in October 1990 marked a significant breakthrough in the field of genetics (5, 6). By the end of this ambitious project, approximately 92% of the human genome was sequenced in April 2003, mainly using Sanger sequencing. The resulting comprehensive genomic map included most of the estimated 20,000–25,000 human protein-coding genes and their organizational structure. However, repetitive and homologous genomic regions were unresolved. In 2022, the telomere to telomere (T2T) consortium published near-complete sequences of all 24 human chromosomes using advanced sequencing methods, identifying 19.969 protein coding genes. (Box 1).

BOX 1 **Take home for clinicians**

Sanger sequencing was the method of choice for genetic routine diagnostics for many years; however, it is relatively labor-intensive and cost-expensive. With emerging next-generation sequencing techniques, it is used less frequently nowadays, mostly only for predictive diagnostics. It still plays a role in confirming unclear findings from Next-Generation Sequencing.

#### 1.1.1 Next-generation sequencing (NGS)

The advent of next-generation sequencing (NGS) technologies in the mid to late 2000s dramatically increased the speed and reduced the cost of DNA sequencing by introducing parallel data generation from usually small DNA fragments (“massive parallel high-throughput sequencing”). This greatly facilitated the sequencing of vast amounts of DNA, enabling the comprehensive analysis of human genomes in diagnostic settings and the identification of genetic variants at an unprecedented scale previously not possible. Short-read (or “second generation”) NGS, emerging in genetic diagnostics since 2005, sequences patient DNA fragments up to 160 base pairs, producing sequencing “reads”. Newer NGS technologies generate up to 20 billion reads within a day, allowing to sequence more than 20 human genomes in a single run. These reads are then compared to a reference genome to detect differences.

Long-read NGS (“third-generation technique”), sequences DNA stretches up to >100,000 base pairs, facilitating complex structural variation analysis. Though currently more common in research due to complexity and cost, decreasing expenses may integrate long-read NGS into genetic diagnostics. However, managing vast data generated, sometimes reaching a terabyte for a single genome, poses challenges in storage, transfer, and analysis, demanding substantial computational resources.

NGS methods (tNGS, WES,) enable cost-effective and swift analysis of multiple genes. tNGS encompasses a certain primer set amplifying a selected group of genes (e.g., 120 primary immunodeficiency genes). This approach offers high-accuracy variant detection but generally does not provide new insights into the role of yet unknown genes. Nevertheless, it can be particularly suitable for the identification of mosaicism due to a high sequencing depth (7). tNGS panels were widely used in the last decade, but many genetic laboratories have switched to whole exome sequencing (amplifying all exons) and then applying virtual “*in silico*” gene panels as filter. This sometimes leads to confusion as the term “gene panel investigation” does not clarify whether a limited set of genes were amplified or whether a WES was performed, but only a limited number thereof analyzed. WES, in contrast to tNGS, enables analysis of almost all genomic protein coding regions, which only represent about 1% of the entire genome but account for about 85% of disease-causing variants (8). WES achieves extensive coverage of coding variants and is useful for the identification of genetic variants in numerous diseases.

Finally, WGS covers almost all genomic regions, including non-coding (intronic) regions and mitochondrial DNA (mtDNA). The non-protein coding portions of the genome correspond to about 99% of the genome. The biological and therefore also clinical evaluation of most variants in non-coding regions is difficult, and often requires extensive functional testing to provide a more definitive assessment of the effects of a variant in these regions. Larger copy number variations (deletions or duplications) that were previously only be detectable by complementary methods such as array-CGH analysis are now reliably detected by WGS.

Different studies focus on evaluating the yield of NGS-based approaches in patients with IEI (9–13), which have been summarized by Vorstefeld et al. (14). The average diagnostic yield of NGS in IEI was found to be 29%, with a range of 10%–70%. For WES, the average yield was 38% (ranging from 15% to 70%), which suggests that in a significant number of cases, NGS-based sequencing approaches such as WES do not effectively diagnose the majority of patients with IEI. Of course, reported percentages expressing a diagnostic yield highly depend on the inclusion criteria, the severity of the phenotype and the depth of the immunological analysis performed prior to genetic testing. However, it is important to highlight that in a considerable number of IEI patients the genetic cause cannot be identified, and a negative genetic test does not rule out the diagnosis of an IEI (Box 2).

The challenge is to choose the right diagnostic tool depending on presentation, suspected (group of) diseases, and available resources: Ideally, genetic analysis identifies a broad spectrum of genetic abnormalities, encompassing not only single nucleotide variations, but structural variants such as duplications, deletions, inversions and translocations in a streamlined process and timely manner. It is also increasingly recognized, that most advanced genetic technologies require interdisciplinary collaborations to achieve the best possible results regarding diagnoses and patient management. Geneticists, immunologists, and clinicians can jointly develop personalized treatment plans that refer to both, the distinct genetic variants and the patient's clinical manifestations.

BOX 2 **Take home for clinicians**

For suspected Inborn errors of immunity (IEI), selecting the most suitable genetic diagnostic method is crucial. Today, routine diagnostic of IEI encompasses WES (sometimes amended by complementary array-CGH in order to address structural aberrations and CNVs) or WGS. This is amended by phenotypical and functional investigations. However, despite the advanced in genetic diagnostics, in many IEI patients, no causative genetic variant can be identified. A negative finding does not rule out the diagnosis of IEI.

### 1.2 Emerging technologies in genetic diagnostics

#### 1.2.1 Optical genome mapping

As in most patients with suspected IEI causative variants cannot be identified by routine NGS methods, further efforts are undertaken with the following research methods:

Optical Genome Mapping (OGM) is a genomic technique detecting various chromosomal rearrangements (like balanced translocations, inversions, and insertions) without constraints of traditional methods such as sequencing or probe hybridization. Unlike short read sequencing (100–160 bp), which struggles in complex regions, OGM achieves full genome assembly using long DNA fragments (150 kbp–1 Mbp). It visualizes DNA fragments tagged with a specific sequence motif (CTTAAG) that acts as a “barcode” for comparison to a reference genome. OGM boasts a whole genome analysis with up to 500 bp resolution, surpassing array-CGH's 20 kb–100,000 kb resolution. While promising for diagnostics, it is primarily used in research due to challenges like DNA quality requirements and complex data interpretation. OGM excels in detecting structural variants (SVs) and copy number variations (CNVs) but cannot identify single nucleotide variants or small indels common in genetic disorders. Combining OGM with other methods, like NGS, may offer a comprehensive view of a patient's genome. OGM holds potential for revealing complete genetic variations in critical immune system-related genes, being especially valuable in these highly polymorphic regions. Some publications concerning other diseases have indicated the benefit of its use for IEI. In Sahajpal et al., OGM has been performed on 57 severely ill COVID-19 patients, and seven SVs have been identified as affecting genes that are involved in innate immunity, inflammatory response, and viral replication and spread (15). These examples underline the potential relevance of OGM in immunodeficient phenotypes, especially because of its superiority in the detection and description of complex variants.

#### 1.2.2 RNA sequencing

Coding variants account for over 85% of pathogenic or likely pathogenic variants in clinical databases (16). Nonetheless, it is widely accepted that non-coding variants also play a significant role in human diseases (17). RNA sequencing (RNA-seq) has emerged as a powerful technique to study gene expression and transcriptomic changes due to non-coding and splicing variants. To date, molecular diagnostic RNA-seq is primarily being used as a research tool. It has been demonstrated to augment the diagnostic yield by approximately 15% compared to WES alone (18, 19). However, selecting an appropriate source (e.g., whole-blood, leukocyte subsets, tissue) for RNA-seq plays an important role in obtaining the optimal diagnostic yield. For example, specific pathogenic splicing variants identified in fibroblast samples have been undetectable in blood samples, indicating the limitations of blood as the sole tissue for certain analyses (20). Furthermore, the analysis of RNA-seq for diagnostic purposes requires normalized samples and a comprehensive control dataset for statistical comparison (21, 22).

In IEI, in which the expression of disease-causing genes is often suppressed, targeted RNA sequencing (T-RNA-seq) is particularly valuable (23, 24). T-RNA-seq focuses on genes of interest, providing exquisite sensitivity for transcript detection and quantification. Numerous studies on IEI have provided compelling evidence for the effectiveness of RNA-seq or T-RNA-seq as powerful tools in the field. For example, intronic variants in the genes *STAT1, DOCK8* and *IL6ST* or in the non-coding gene *RNU4ATAC* have been shown to be pathogenic using RNA-seq (25–28).

#### 1.2.3 Proteomics

Over the past two decades, mass spectronomy-based (MS-based) proteomics has provided significant advances in the field of immunology (29). High-resolution mass spectrometry is a powerful method for profiling and quantifying proteins in tissues, organs, and cells, enabling comprehensive exploration of cellular processes, signaling pathways, post-translational modifications, and protein interaction networks (30). This approach has enhanced our understanding of the dynamic and complex nature of the immune system, shedding light on its functioning and underlying mechanisms. A recent review of the literature has highlighted the significant contributions of MS-based proteomics to our understanding of innate immunity (31).

Proteomics has been employed in a limited number of studies for the genetic diagnosis of IEI (32, 33). Despite the impressive biological insights provided by MS-based proteomics its integration into mainstream diagnostic laboratories is limited by cost and lack of expertise in data analysis and interpretation.

## 2 Further emerging diagnostic genetic approaches

Besides OGM, RNA-seq and proteomics, other genetic approaches, such as single cell sequencing, epigenomics, metabolomics or multiomics are increasingly relevant in both research and diagnostics. These aspects are briefly discussed here but are beyond the scope of this review.

Single cell sequencing allows the analysis of individual cells to identify their genetic profile, which is particularly useful in heterogeneous cell populations such as immune cells, and could play a crucial role for understanding and diagnosing IEIs (34, 35). It can be divided into single cell DNA (scDNA-seq) and single cell RNA sequencing (RNA-seq), belonging to single-cell genomics and single-cell transcriptomics (36), respectively. One of the advantages of scDNA-seq over bulk DNA sequencing is the higher sensitivity of mosaicism detection. Increasing the depth of bulk DNA sequencing does not eliminate the risk of missing mosaic features, as mosaicism with less than 0.5% cannot be distinguished from sequencing error (36). In addition, single cell analysis allows to further define low-level mosaic variants detected by bulk sequencing and determine their origin (i.e., the same cell or to different cells). Besides, it allows the association of a genetic feature with the phenotypic character of a specific cell type. The review by Evrony et al. (36) gives an overview on the major applications of scDNA-seq.

In the context of IEIs, the importance of understanding the interplay between genetic and epigenetic factors such as DNA methylation, chromatin remodeling, and histone acetylation are crucial. The differentiation of immune system cells relies on the presence of a DNA methylation pattern. Any dysfunction or impairment in the DNA methylation machinery may lead to immune dysfunction and the onset of various diseases. This is excellently summarized in a review by Romano et al. (37). Hypermethylation of genes like *PIK3CD*, *BCL2L1*, *RPS6KB2*, *TCF3* and *KCNN4* and the decreased ability to demethylate them led to an impaired transition from naive to memory cells shown in a study on CVID-discordant monozygotic twins (38). Moreover, in cohorts of CVID-patient and healthy controls, different methylation patterns of relevant genes of B-cell development and function could be observed (39). The immunodeficiency, centromeric instability, facial anomalies syndrome, type 1 (ICF1) can be caused by mutations in *DNMT3B*. The impaired function of this gene leads to changed methylation of regulatory regions of lineage-specific immune-related genes during development which cause the phenotype of ICF1. Correction of *DNMT3B* variants using CRISPR-Cas9 could partially restore the healthy epigenome (40).

**Figure 1:**
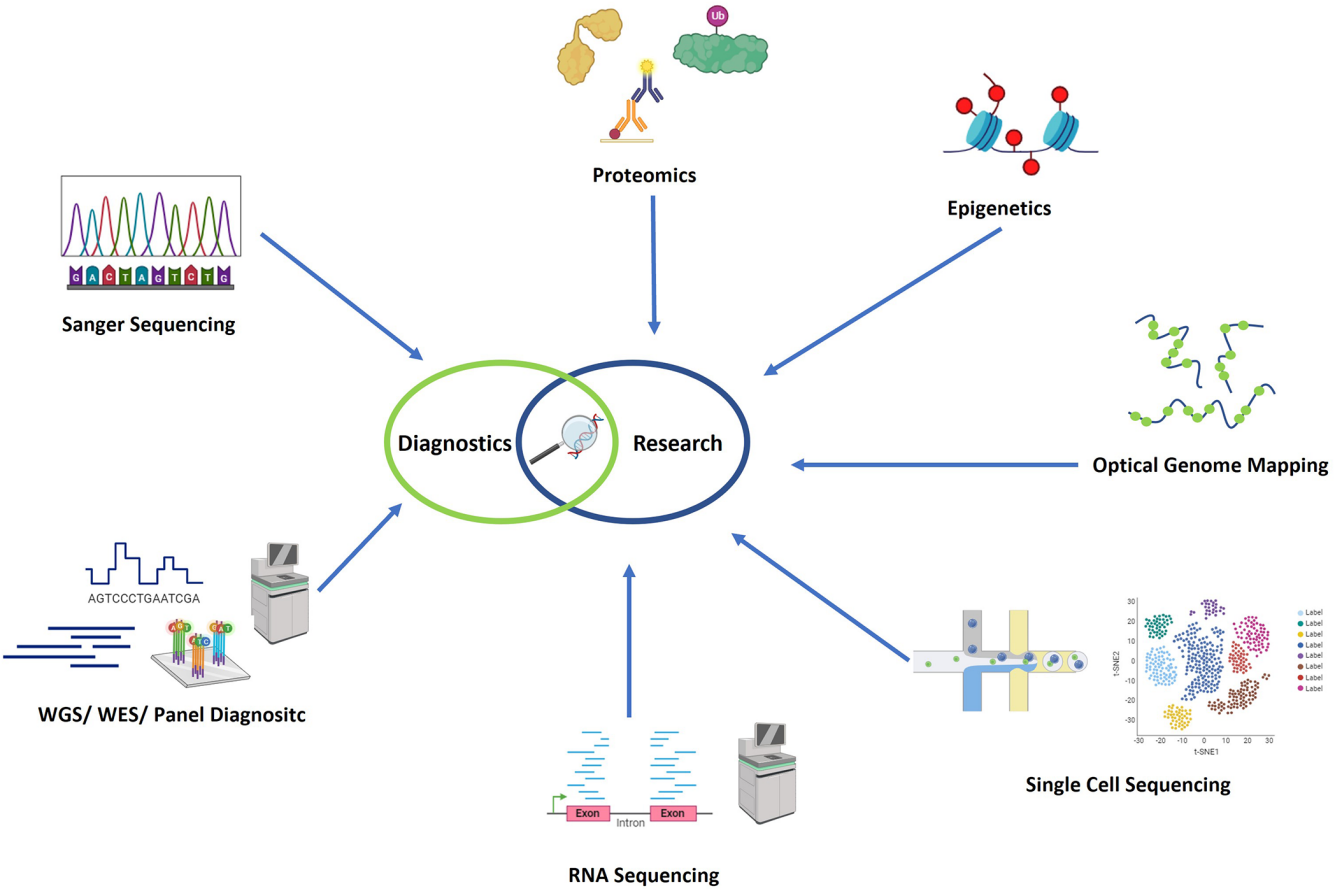
Overview of the most commonly used genetic technologies. In the field of genetic diagnostics, Sanger sequencing, panel diagnostics, whole exome sequencing, and whole genome sequencing have recently been employed as technologies. Additionally, in research settings, optical genome mapping (OGM), RNA sequencing (RNA-seq), proteomics, single cell sequencing, and epigenomics are additionally used in research settings. Figure created with Biorender.com.

Most likely, to provide a holistic view of the molecular basis of diseases, a multiomics approach is preferred. This would include the above-mentioned genomics, transcriptomic, proteomics, metabolomics, epigenetics and other “omics” data. Chu et al. and others provide overviews of the various methodological approaches available for the different omics data layers that are relevant in immunological research (41, 42). In Figure 1 an overview of the most commonly used genetic technologies is given.

### 2.1 Analytic strategy of genomic data of patients with IEI

The ACMG/AMP (American College of Medical Genetics and Genomics/Association for Molecular Pathology) classification system was established for the evaluation and classification of sequence variants for Mendelian diseases based on single gene defects (43). This system recommends the use of a specific standard terminology: pathogenic (class 5), likely pathogenic (class 4), unknown significance (class 3), likely benign (class 2), and benign (class 1) (Table 1). To classify a sequence variant, several criteria are used, including the type and location of the variant, frequency in the general population, listing in gene-specific databases, evaluation by bioinformatic prediction programs, and segregation within the family. The use of the ACMG/AMP classification system has become increasingly important in clinical practice to guide patient management and counselling, and to improve the accuracy and consistency of variant interpretation. Clinical consequences are currently recommended only for class 4 and class 5 variants (Table 1).

**Table 1:** Standard terminology of the ACMG/AMP classification system.

ACMG classification	Class	Probability of pathogenicity clinical	Consequences
Pathogenic	5	>99%	e.g., diagnosis, prognosis, therapy as well as testing of family members
Likely pathogenic	4	>90%	
Variant of unknown significance (VUS)	3	10 bis 90%	Currently none, also no testing of family members
Likely benign	2	<10%	No communication, no consequences
Benign	1	<0.1%	

### 2.2 Gene panels based on the IUIS classification of IEI

In 1973, the International Union of Immunological Societies (IUIS) Committee was established by the World Health Organization with the primary objective of characterizing and categorizing IEIs in humans. Since then, a curated list of immunologic disorders has been authored by the committee, ensuring a standard nomenclature and consistent approach. Following the discovery of genetic defects associated with IEI, the committee has begun to include a list of genes linked to IEI in peer-reviewed publications. This list is updated every two to three years. In October 2022, the most recent update has been released, which includes 485 genes linked to IEI, including 55 additional genes since the 2019 IUIS update (44). These genes are divided into ten categories (45) (Table 2). The implementation of these categories into routine diagnostic as defined *in silico* gene panels would allow the efficient and accurate analysis of genes associated with specific IEI groups. However, there is no unified procedure for this. In the Netherlands, an identical *in silico* gene panel with 389 genes for IEI is used nationwide (46) whereas diagnostic *in silico* panels used in other countries and centers differ across laboratories. As there is also a worldwide standardized classification system of detected variants in genes associated with IEI, the use of a standardized *in silico* gene panel should be recommended. This would also prevent the large variability in diagnostic yield, which has been reported with an average of 38% (with a range of 15%–70%) in context of IEIs (14, 47, 48).

**Table 2:** Gene panel based on the IUIS classification of inborn errors of immunity.

Table	Genpanel based on the IUIS classification of genetically inherited immunodeficiencies
1	combined immunodeficiencies
2	combined immunodeficiencies with syndromic features
3	predominantly antibody deficiencies
4	diseases of immune dysregulation
5	congenital defects of phagocytes
6	defects in intrinsic and innate immunity
7	autoinflammatory diseases
8	complement deficiencies
9	bone marrow failure
10	phenocopies of inborn errors of immunity

### 2.3 Gene panels based on the clinical genome resource (ClinGen)

The Clinical Genome Resource (ClinGen) is a collaboration between US-American National Institutes of Health (NIH), academic institutions, and industry partners with over 2,200 contributors from more than 62 countries. It was funded in 2013 to promote the knowledge on clinical relevance of genes and variants for use in precision medicine and research. ClinGen has established several working groups focused on specific rare disease areas, such as neurodevelopmental, cardiovascular, neurological or immunological disorders. These Clinical Domain Working Groups bring together experts from different fields to evaluate the strength of evidence of gene-disease relationships and create a gene curation expert panel. The Clinical Domain Working Group “Immunology” curates clinically relevant and actionable genes causative for diseases of the immune system. To date, the gene curation expert panels for antibody deficiencies, primary immune regulatory disorders and SCID-CID (severe combined immunodeficiency-common variable immunodeficiency) are completed and publicly available (https://www.clinicalgenome.org/working-groups/).

### 2.4 Human phenotype ontology (HPO) based analysis

The Human Phenotype Ontology (HPO) is a standardized description of human phenotypes, emphasizing those seen in genetic disorders (49, 50). Each HPO term details a specific abnormality in human traits linked to genes causing diseases defined by OMIM (51). With over 13,000 terms, HPO is crucial for analyzing clinical WES and WGS data. Bioinformatics tools integrate an individual's HPO-coded phenotype with sequencing data to prioritize causal genes.

Despite its utility, applying HPOs in clinical practice presents challenges. Patients exhibit not just disease-specific symptoms but also secondary signs shared by various conditions. Moreover, unrelated medical issues may confound diagnosis. Limited availability of comprehensive IEI-related HPO terms hinders its widespread use (52, 53). In genetic testing for IEIs, tools using HPO terms failed to identify disease-causing genes in 37% of patients with monogenic disorders (12).

Efforts from the ESID genetics working party and ERN-RITA aim to refine IEI-related HPO terminology. Haimel et al. have enhanced the HPO vocabulary by generating more comprehensive sets of terms specifically related to IEIs. They have thoroughly examined four distinct branches of the HPO tree, contributing a total of 57 newly developed and extended terms to the HPO. The majority of these terms has been successfully incorporated into the official HPO data set (53).

### 2.5 ESID classification

ESID, established in 1994, aims to advance knowledge on IEIs through education, research, and best practice guidelines. Its continuously updated registry, launched online in 2004, gathers clinical and research data of IEI patients globally. ESID's “working definitions for clinical diagnosis of primary immunodeficiencies” help diagnose and register IEIs based on standardized criteria, facilitating global communication among scientists and physicians. These criteria categorize immune system disorders (e.g., T-cell deficiencies, B-cell deficiencies), enabling comprehensive understanding and data organization. The criteria consider both clinical and laboratory characteristics, aiding in pattern recognition within disorders for improved IEI management, genetic testing recommendations, and further studies. However, they must be applied considering individual patient characteristics and clinical context. As research evolves, these criteria may require updates to reflect new classifications or insights.

## 3 Comprehensive approaches for analysis of genomic data

### 3.1 Family based sequencing

Simultaneous genetic analysis of the patient and their parents [NGS-based Trio (Trio WES or Trio-WGS)] is a useful approach to speed up the process of making a precise genetic diagnosis (54). This is because the parental data and segregation information for each variant are immediately available, facilitating clinical interpretation of the variants. This can be of particular importance in case of severely ill infants who are admitted to neonatal or pediatric intensive care units or for patients who benefit from precision treatments (e.g., patients with SCID and life-threatening infections in infancy). Furthermore, NGS-based Trio analysis allows the reliable detection of *de novo* variants without the addition of further analysis, which leads to a faster turnaround time and a higher detection rate. Farwell et al. have estimated Trio analyses to have a diagnostic yield of 37%, compared to 21% for single gene analyses (45). Identification of potential new disease-causing genes is also more likely with Trio analysis. There are also a few possible contraindications or disadvantages associated with NGS-based Trio analysis. (1) Cost: NGS-based Trio analysis involves sequencing of three individuals, which is more expensive compared to individual WES or WGS. The increased cost may be a limiting factor, especially in situations where financial resources are limited. (2) Ethical concerns: NGS-based Trio analysis raises ethical questions, particularly when it comes to obtaining informed consent from all individuals involved. It is important to ensure that all patients understand the implications of NGS-based Trio analysis, including the potential identification of genetic conditions or predispositions that might have consequences for the whole family. (3) Privacy concerns: NGS-based Trio analysis involves the analysis of genomic data from multiple individuals within a family, raising privacy concerns. Obtaining comprehensive clinical information and medical history of all individuals undergoing sequencing is essential for meaningful data analysis. (4) Data interpretation: In the process of analysis strategy, it is crucial to consider the presence of variants with variable expressivity and incomplete penetrance within the family. Even if a genetic variant is identified in the individual, it may not necessarily present with the associated condition or disease. Alternatively, even if a genetic variant is inherited from a parent, it may not necessarily be excluded to be causative. This can lead to challenges in determining the clinical significance of the variant. In any case, it must be ensured that the results are interpreted in the context of the individual's clinical and family history.

Certainly, this issue also has to be considered if prenatal testing is an option for affected families. Especially, in the context of genetic alterations with variable clinical expressivity and incomplete penetrance, uncertainty may arise regarding the actual impact of the alteration on the health and development of the unborn child. Overall, navigating the ethical complexities of prenatal diagnosis involves a careful balance between providing parents with the information they need to make informed decisions and respecting their autonomy, all while acknowledging the uncertainties inherent in genetic medicine. Therefore, genetic counselling is an integral part of prenatal testing in families with inborn errors of immunity. However, particularly in families with IEI, early diagnosis can be instrumental in saving lives. Therefore, it is valuable to ascertain, even in unborn children, whether they are highly likely to be affected by an IEI.

### 3.2 Genematcher approach

**Figure 2:**
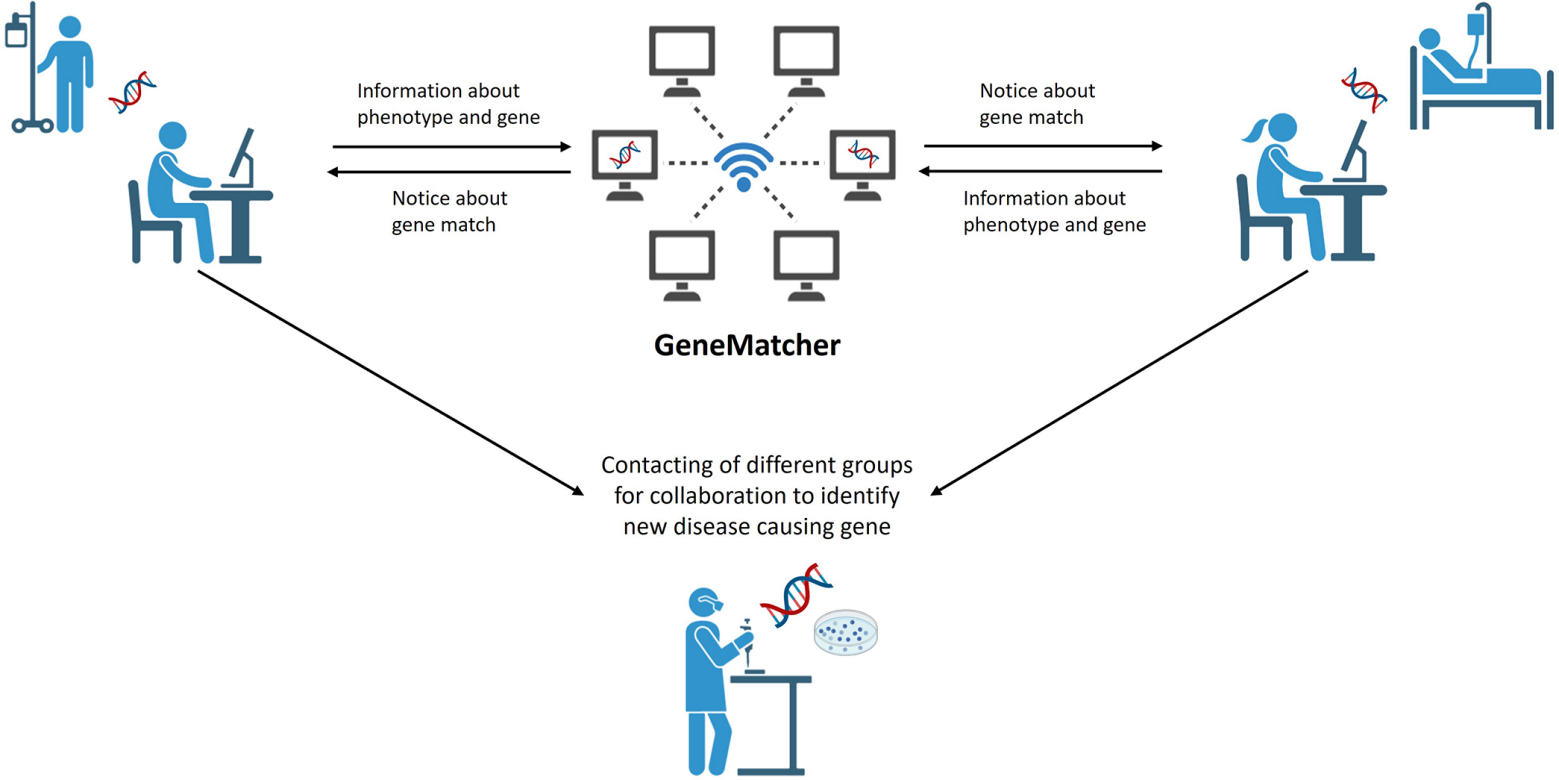
Genematcher approach. Web based tools like GeneMatcher are used to share information about phenotypes of patients with IEI and the results of WGS analysis. GeneMatcher informs researchers about a “match”—same gene was uploaded by others—that allows them to get in touch, to exchange about phenotypes and results and to collaborate for further analysis of potential disease-causing genes. Figure created with Biorender.com.

NGS-based Trio analysis has played a significant role in expanding our understanding of rare diseases by identifying new disease-causing genes. Web-based tools enable scientists from around the world with an interest in the same genes, variants or phenotypes to collaborate (e.g., GeneMatcher—https://genematcher.org/, Variant Matcher—https://variantmatcher.org/, phenodb—https://phenodb.org/). These collaborative approaches allow researchers to pool their resources, expertise, and patient data, leading to more robust and comprehensive analyses. This enhances the statistical power and accuracy of genetic studies, ultimately increasing the chances of finding disease-causing variants and improving patient outcomes. Through these collaborations, ideas are exchanged, and valuable methodologies are shared (Figure 2). Researchers and physicians worldwide benefit from each other's discoveries, ultimately hastening the pace of research. To assess the impact of these collaborative approaches is challenging; however, the significance is evident through the number of matches and publications they facilitate. For instance, since 2015 GeneMatcher has been cited in 753 publications (https://genematcher.org/statistics/).

Different studies have also demonstrated the advantage of using GeneMatcher in IEI (55–61). An international team, for instance, was able to identify five families with ten patients exhibiting a similar constellation of symptoms, including medically refractory infantile-onset inflammatory bowel disease (IBD), bilateral sensorineural hearing loss and, in most cases, recurrent infections. All patients carry biallelic or monoallelic damaging variants in *STXBP3*. Through GeneMatcher three families with immune-associated defects, poor growth, pancytopenia and skin pigmentation abnormalities have been ascertained. All affected patients carry biallelic *DPP9* rare variants. Another international team, connected through GeneMatcher, has identified a total of 15 patients from eight families to have an autosomal recessive immunodeficiency syndrome characterized by severe infections caused by both RNA and DNA viruses, along with virally triggered inflammatory episodes associated with hemophagocytic lymphohistiocytosis-like disease. These patients also presented with early-onset seizures, as well as renal and lung disease. All of them carry biallelic damaging variants in *ZNFX1*.

### 3.3 Artificial intelligence and machine learning in genetic diagnostics

Artificial Intelligence (AI) and Machine Learning (ML) tools have demonstrated considerable potential in genomics research. Notable examples include facial analysis for genetic disorder identification and machine learning for variant classification or risk-assessment algorithms. AI programs, such as Face2Gene (https://www.face2gene.com/) have emerged as a valuable aid by analyzing facial features to assist in the diagnosis of specific genetic conditions, potentially improving early detection and patient management. For pediatrician's clinical workflow, the Face2Gene platform has introduced a specialized feature known as the “Pediatrician View.” This functionality facilitates the analysis of patients by incorporating facial images. Upon uploading a portrait photo, the system computes a normalized score termed the facial D-Score. The facial D-Score serves as an indicator of the likelihood of dysmorphic features being present in the patient's photo. A higher D-Score corresponds to a higher probability of the existence of such features. This scoring mechanism can assist clinicians in making informed decisions about whether a patient should be referred for further genetic diagnostic evaluation (https://www.face2gene.com/pediatrician-view/). Furthermore AI-based phenotypic scores of facial image data, generated by Face2Gene, GestaltMatcher, Phenoscore etc., could be implemented into variant classification (62). ML algorithms are also being developed to distinguish pathogenic genomic variants from benign ones (63). These deep learning networks predict the pathogenicity of genetic variants from curated datasets and various genomic features, including experimental, population and clinical data, thereby assisting in the interpretation of genetic testing results. Mostly, an automated, streamlined process identifies a concise list of candidate genes for comprehensive evaluation, and reporting (64, 65). The automation of genetic disease diagnosis potentially simplifies and expedites the interpretation of the vast numbers of genetic variants, leading to an increased diagnostic yield while reducing turnaround time and cost. Different studies have already shown a benefit in using these tools (64). A recent publication has presented a prospective cohort study that has successfully validated an ML approach for risk stratification of IEI. This approach exploits ICD codes extracted from electronic health records to discriminate between datasets linked to children diagnosed with IEI and those without. The approach has demonstrated an accuracy rate of 89% in diagnosing patients with IEI (66). Despite all the benefits using AI and ML in genetic approaches, it is important to be cautious about biases as the effectiveness of algorithms depends on the quality of the training data. Recognizing that algorithms are developed by humans with biases and individual perspectives underscores the need for caution.

## 4 Current challenges

With the vast volume of data generated by NGS, the importance of effective filter techniques cannot be overstated. These are necessary to reduce the multitude of identified variants to a manageable subset of potentially clinically relevant ones. Appropriate filtering strategies consider factors such as variant frequency in population databases, predicted functional impact, inheritance pattern, and consistency with the patient's phenotype. VUS pose a particular challenge, as their impact on protein function and contribution to disease phenotype is uncertain. Novel bioinformatics approaches are continually being developed to predict the potential pathogenicity of these variants, employing machine learning and integrating diverse data types. In particular, when using WES or WGS as a diagnostic method, the possibility exists of identifying pathogenic variants in genes that are not associated with the actual diagnostic request, so called “incidental” or “secondary” findings. Before conducting extensive genetic diagnostics, it should always be clarified between the patient, the requesting physician, and the performing laboratory how to handle incidental or secondary findings. The implementation of accurate filters can minimize the likelyhood of incidental or secondary findings, thus mitigating potential ethical implications. It is worth noting that no filtering strategy is perfect, and rare pathogenic variants can be incorrectly filtered out, stressing the need for continual refinement of these methods based on the latest research findings. Therefore, establishing robust and accurate filter techniques is fundamental to harness the power of NGS in the diagnostics of IEI, striking a balance between sensitivity and specificity to ensure that relevant pathogenic variants are detected while limiting the identification of irrelevant ones.

While technological advances in sequencing and bioinformatics play a significant role in the process of establishing genetic diagnosis, the human factor remains a critical component in the interpretation and application of these results. Genetic diagnostics should ideally be performed in specialized centers with experienced human geneticists who have a deep understanding of IEI genetics. These professionals bring the necessary capacity to integrate complex genetic data with clinical information, including the patient's symptoms, family history, and laboratory findings, to provide a meaningful interpretation of sequencing results and enabling or arrange functional diagnostics in unclear cases. A nuanced understanding of IEIs can enable geneticists to anticipate and recognize atypical presentations and variable expressivity of diseases, to consider the impact of genetic modifiers, and to factor in potential non-genetic causes. Furthermore, they can give guidance on the follow-up functional studies needed to validate the impact of novel variants and to correlate genotype with phenotype. Of note, the geneticists depend on the clinicians who ideally provide clinical and phenotypical information as detailed as possible. Geneticists in these specialized settings can also play a pivotal role in communicating complex genetic information to patients and their families, helping them understand the implications of genetic diagnoses for disease prognosis, management, and family planning. Thus, while we continue to automate and refine our technical capabilities, expertise and judgment of human geneticists remain invaluable in the field of IEI genetic diagnostics.

### 4.1 Dealing with variants of unknown significance (VUS)

As diagnostic genetic sequencing becomes more comprehensive, the frequency of detecting variants that cannot be classified as either benign or pathogenic, referred to as VUS, is also increasing. A VUS is defined as a variant with an unclear or unknown association with disease risk. In many cases, these variants are very rare in the population so that there is limited information available about them. Additional data (e.g., further phenotypical or functional analyses) are usually required to evaluate its pathogenicity. However, these analyses are not typically performed as part of genetic diagnostics. The detection of VUS can create uncertainty for treating physicians and patients alike, raising questions such as whether the disease's underlying cause has been identified and whether additional analyses are necessary. In order to preemptively alleviate uncertainty for patients and their parents, it is of paramount importance to inform patients during the genetic diagnostic request that there may be findings involving VUS, and that every individual, including healthy individuals, may harbor a number of VUS. It is generally advised not to base clinical recommendations on the presence of a VUS andthe 2015 ACMG/AMP variant classification guidelines state explicitly that a VUS should not be used in clinical decision-making. When a patient is found to have a VUS, any clinical decisions should rely on their individual and family history rather than the presence of the VUS. Genetic analysis of parents or other family members may generate additional evidence for a potential VUS reclassification. The classification of a VUS may evolve over time. Therefore, it is equally important to request a reassessment of a dataset after a specified period, such as two years, to allow for the possibility of a more certain diagnosis through changes in interpretation.

Furthermore, it also may be of importance to functionally validate a variant classified as pathogenic if the variant does not explain the reported phenotype. These variants normally should not be reported by genetic laboratories but such variants, however, could be discussed with clinicians when there are doubts about the specific phenotype of the patient. Nevertheless, it is predicted that a significant number of VUSs in coding regions will be elucidated by 2030. This assumption is driven by the progress in standards for variant classification, the enhancements in the performance of computational variant effect predictors, the scalability of multiplexed assays capable of thoroughly examining variant effects across the genome, and collaborative data-sharing initiatives poised to extract maximum information from each newly sequenced individual and interpreted variant (67). In the majority of cases, immunological functional testing in patients does not result in in a change of classification of the genetic variant. For instance, neutrophil granulocyte dysfunction cannot be employed to reclassify a VUS in the CYBB gene as (likely) pathogenic. This is due to the possibility of a different, unidentified variant in the same gene or another variant in a different gene being responsible for the observed phenotype. To use functional analyses for reclassification purposes, it is essential to unequivocally demonstrate through the analysis that the variant under investigation distinctly leads to an altered function of the gene or the gene product.

### 4.2 Challenges in analysing gain-of-function (GoF) variants

The phenotypic expression of many genetic variants can vary significantly, especially in IEI, exhibiting variable expressivity, and the development of disease may not occur with 100% certainty (reduced penetrance). Furthermore, in recent years, there has been a significant increase in the identification of variants that result in a hypermorphic or neomorphic gain of function (GoF) effect. These variants lead to an enhanced or entirely new protein function. The identification and classification of GoF variants remains a challenge, even for geneticist, because prediction algorithms for determining pathogenicity of GoF variants are not reliably usable and ACMG criteria do not apply well. Therefore, regardless of the prediction algorithms used, both the phenotype and pedigree of the patient, as well as the function of a gene, play crucial roles in interpretation of variants. A practical guide for WES analysis is given by Vorsteveld et al. (14).

In some genes, both disease causing loss of function (LoF) and GoF variants have been reported. In the *STAT3* gene, GoF variants lead to its hyperactivation, causing immune dysregulation, early-onset lymphoproliferation and autoimmunity (68) whereas LoF variants result in impaired *STAT3* function, leading to a hyper-IgE recurrent infection syndrome-1 (HIES1) (69, 70). Another puzzling feature that seems to be particularly frequent in genes associated with IEI is the observation that pathogenic variants of the same gene can follow different modes of inheritance. For example, both an autosomal recessive as well as an autosomal dominant inheritance is known to be causative in the genes such as *MEFV* (71, 72), *STING1* (73, 74) and *AICDA* (75, 76).

### 4.3 Somatic variants and mosaicism

IEI are most often caused by germline variants—genetic alterations that are present in every cell of the body. However, recent research has uncovered a significant role for somatic or post-zygotic variants—those that arise in a cell during the course of an individual's life and are not present in every cell—in these disorders. Somatic variants can lead to a mosaic pattern of disease, where some cells in the body carry the variant and others do not. These mosaic disorders can often present with atypical or milder phenotypes compared to their germline counterparts due to the presence of a population of normal cells. Several immune disorders have been associated with somatic variants.

Phenocopies refers to a category of disorders that exhibit clinical manifestations similar to IEI. However, in the case of phenocopies, the observed clinical features mimic those of IEIs without an underlying genetic defect. Instead, these disorders may be caused by somatic variants or other non-genetic factors (e.g., autoantibodies against various cytokines), leading to a phenotypic similarity to IEIs (45, 77). These disorders do not adhere to a Mendelian pattern of inheritance and the IUIS has designated phenocopies of IEIs as a distinct classification.

The identification of somatic variants using NGS demands specialized filters and algorithms due to the occurrence of these variants at exceptionally low allele frequencies (AF). The AF represents the proportion of mutated alleles in the sample. The AF for a somatic variant is influenced by the heterogeneity of the chosen tissue or sample for sequencing (78).

For example, somatic variants in the FAS-pathway cause autoimmune lymphoproliferative syndrome (ALPS). Other examples are autoinflammatory diseases such as AIFEC (autoinflammation with infantile enterocolitis) or NOMID (neonatal onset multisystem inflammatory disease) due to mosaicism in *NLRC4* in young children (79) or VEXAS (Vacuoles, E1 enzyme, X-linked, Autoinflammatory, Somatic syndrome) due to *UBA1*-variants in the elderly (80). Detecting these somatic variants requires sensitive techniques as the “mutated” cells may be a small proportion of the total blood cells in the body. The recognition of somatic variants in IEI has important implications for diagnosis and treatment, as well as for genetic counselling of affected individuals and their families.

## 5 Early genetic diagnosis is crucial for optimal treatment

An early molecular diagnosis of IEI is associated with improved health outcomes, decreased healthcare costs, and mitigates psychological stress for affected families (81–83). According to the ESID data, there is a 1.7% increase in the risk of mortality for each year of delay in diagnosis (84). Moreover, a genetic diagnosis paves the way for fundamental therapies in 34% (85), specifically for Hematopoietic Stem Cell Transplantation (HSCT), which is most effective when initiated early in the disease course before significant damage to the affected organs ensues (81). However, available therapies have been progressively expanding to include small molecule inhibitors, biologicals, gene therapy, and the use of adoptive transfer of virus-specific T cells to combat viral infections in immunocompromised patients (86). The rarity of the individual immunological diseases makes it difficult to conduct controlled studies, highlighting the necessity of thoroughly understanding the immunologic aetiology and possibly the underlying genetic causes to develop feasible hypotheses about how regulation of the immune response would affect the clinical course of the disease. As a result, genetic testing has become an indispensable tool for diagnosing and managing children afflicted with IEI (48).

## 6 Concluding remarks

In recent years, there has been an exponential increase in knowledge in human genetics, primarily driven by the development of new investigative techniques. This advancement has benefited many other disciplines dealing with the treatment of rare diseases. Although most IEIs are monogenic, many exhibit variable expressivity and penetrance, and reliable genotype-phenotype correlations are lacking. This emphasizes the importance of genetic diagnostics, which is becoming increasingly crucial in facilitating the diagnosis of these disorders.

Therefore, standardization is required in clinical practice by considering the clinical and laboratory characteristics of the patient when deciding on the genetic testing method. Close collaborations between physicians and geneticists are required to ensure on the one hand an efficient workflow to identify potential pathogenic variants that may have a significant impact on further therapies, especially for severely affected patients. On the other hand, even though prompt decision-making is essential, a prudent examination of variants should be pursued, and there may arise situations where it becomes necessary to engage specialized laboratories to conduct functional analyses, which, in turn, consume additional time. Finding the balance between efficient and rational use of all the modern testing methods is one of the main tasks for physicians and geneticists. Although various AI-driven tools are now available supporting the decision whether genetic testing is helpful, interpreting genetic data is far from straightforward and bears the risk of misinterpretation. Therefore, it is advisable to determine which genetic laboratory is specialized in IEI before initiating genetic diagnostics.
